# Paternal Social Determinants of Childhood Mortality in Zimbabwe

**DOI:** 10.5334/aogh.4591

**Published:** 2024-12-24

**Authors:** Laston Gonah, Dumisani Sibanda, Sibusiso C Nomatshilla

**Affiliations:** 1 Department of Public Health, Faculty of Medicine and Health Sciences, Walter Sisulu University, South Africa

**Keywords:** Social determinants of health, paternal determinants, under‑five‑year mortality, child mortality, infant mortality, survival analysis, Zimbabwe

## Abstract

*Introduction:* In Zimbabwe, studies have mainly focused on child and maternal factors contributing to under‑5‑year mortality, and little has been published concerning the paternal social determinants, which are also important.

*Aim:* The goal of this paper is to investigate the paternal social determinants of infant and childhood mortality in Zimbabwe.

*Methods:* The study analyzed cross‑sectional secondary data from the Zimbabwe Demographic Health Survey (ZDHS) 2015 to investigate paternal determinants of infant and child mortality. Multivariate logistic regression and Cox regression were conducted for separate analyses of infant and child data to determine the odds and risk of death informed by paternal factors. Kaplan–Meier survival curves were used to determine the importance of paternal factors in determining under‑five survival.

*Results:* Younger paternal age, lower level of education, lower wealth index, unemployment, and rural geographical location are important contributing factors for childhood mortality, and these factors were found to be interconnected and interrelated in producing the observed outcomes.

*Conclusion:* Paternal characteristics are important contributing factors for child survival, but not alone. The interaction between child characteristics, household (paternal and maternal), community, and public/global‑policy‑level factors is important in shaping observed childhood mortality outcomes. Social determinants for child survival are interlinked and interdependent on each other in producing the observed childhood mortality outcomes, and no one factor is more important than the other. Each factor represents an important component but not one that is individually sufficient to produce an outcome.

## Introduction

Infant and child mortality have been mitigated in some other parts of the world; however, some regions continue to have unacceptably high rates [1]. Infant and childhood mortality is a crude marker for the level of socioeconomic development of a country and was found to be sensitive for comparability in different contexts [2]. The greatest burden of infant and child mortality is in sub‑Saharan Africa [1]. Zimbabwe is one of the countries experiencing the highest rates of under‑5‑year mortality (U5MR), as evidenced by failure to meet the previous millennium development goal 4, which sought to reduce under‑5‑year mortality rate (U5MR) to below 27 per 1,000 live births. Zimbabwe’s estimated U5MR in 2015 was 69 per 1,000 live births [3]. The country was ranked among the 50 worst countries for early childhood mortality despite concerted efforts to improve infant and child survival [3]. Zimbabwe has an estimated total population of 13.3 million, translating to a population density of 30 people per km^2^ as of 2007. The population consists of 41% under the age of 15 years, of which 13% (1,706,000) are children under the age of 5 years [3].

Health outcomes such as childhood survival outcomes are known to be caused by the conditions in which children are born and live, including individual and family‑level factors (birth characteristics, parental income, age, level of education, race, socioeconomic position), health system factors, and socioeconomic and political context at national and global levels [4]. These social and economic conditions in which people live that influence their health outcomes are called the social determinants of health. Child survival outcomes were shown to be different between and within countries due to the interaction of these determinants, where high‑income countries are more likely to have stronger health systems and better governance structures and macroeconomic policies for promoting good health and well‑being, thereby resulting in better survival outcomes compared with low‑income countries [4, 5]. Within a country, individual and family‑level factors are important in shaping childhood survival outcomes. These factors such as birth characteristics and maternal determinants have been explored, consistently showing that childhood mortality is, or poor childhood survival outcomes are, linked to poor maternal characteristics. Childhood mortality has been linked to low levels of education, low‑income levels, and younger age on the mother’s part, whereas better maternal characteristics are associated with better chances of childhood survival [4–6].

Maternal determinants of infant and childhood mortality have largely been studied in the Zimbabwean context, and findings have been used to guide the implementation of various interventions across the country [7–9]. However, recent evidence from elsewhere interestingly suggests that paternal factors may also play an important role in modulating infant and childhood mortality, yet these are under‑explored in Zimbabwe [10]. This indicates that determinants of infant and child mortality are driven by several other important factors, apart from maternal‑dependent factors alone. This research aims to study the paternal determinants of infant and childhood mortality whilst controlling for maternal sociodemographic factors.

## Methodology

### Data source

The study utilized cross‑sectional secondary data obtained from the Zimbabwe Demographic Health Survey (ZDHS) that was conducted in 2015 [3]. The ZDHS has become a reliable source of national demographic and health status data for research and formulation of public policy. The primary objective of the 2015 ZDHS survey was to provide estimates for basic demographic and health indicators [3]. The ZDHS collects information on fertility levels, marriage, sexual activity, fertility preferences, awareness of and use of family planning methods, breastfeeding practices, nutritional status of mothers and young children, early childhood mortality, maternal mortality, maternal and child health, knowledge and behavior related to human immunodeficiency virus (HIV)/acquired immunodeficiency syndrome (AIDS) and other sexually transmitted infections (STIs), smoking, knowledge of cervical cancer, and male circumcision [3].

Data for the survey were collected from July to December 2015 from 11,000 households. Women aged 15–49 years and men aged 15–54 years were targeted as eligible respondents from the selected households. The specified population covers the childbearing age range, and the participants are expected to be most likely taking part in the requisite economic and social activities, thereby supplying the adequate information required by the survey.

Access to the ZDHS data was obtained by consent from ICF Macro, the technical lead organization in demographic health surveys.

### Data preparation and variable recoding for analysis

Data from the survey were available in two different datasets, that is, for males and females. The first step was to merge the two datasets in STATA. Merging was done by generating unique identifiers using cluster number, household number and respondent line number from the men’s dataset, and cluster number, household number and line number of the husband from the women’s dataset. This merging ensured that a husband and a wife were matched, and therefore, subsequent analyses were done for infants and children of matched husbands and wives.

Matching was necessary to complement information obtained from the male partner with that from their corresponding female partner, since some of the paternal‑related factors collected from female partners concerning their husbands did not exhaust the paternal factors required for this study, such as use of tobacco and alcohol products by their husbands. Matching also ensured that only those households with a father as part of the household were included. Matching also allowed for corroboration of data collected between the male and female partners to ensure data accuracy since some of the data collected we reprone to recall bias.

Variables from the merged dataset were recoded, and new requisite variables were generated. A new categorical variable was created to categorize the children as neonates, infants, children, and older children. Children older than 60 months were then removed from the dataset to create a new trimmed dataset of children under 60 months belonging to matched husbands and wives. Most of the variables from the original dataset were recoded to generate covariates required for the analysis of this study. The employment categories were reduced to four employment categories, namely unemployed, white collar, blue collar, and other forms of work (e.g., voluntary work, artist work). This was based on findings from other studies that found that these four employment categories can best characterize employment categories that can potentially affect infant and child mortality. The fathers’ religion was also collapsed into traditional, apostolic, Christian, and other religions, which included nonreligious men.

### Sample design

The sample was selected primarily with the goal of yielding a nationally representative sample of demographic and health indicators from across all the country’s eight provinces: Manicaland, Mashonaland Central, Mashonaland East, Mashonaland West, Matebeleland North, Matebeleland South, Midlands, Masvingo, and the two metropolitan provinces of Harare and Bulawayo. The sample was selected using the two‑sample cluster design method. The first stage used enumeration areas as sampling units based on the Zimbabwean map and results of the 2012 population census. The first stage of sampling yielded 400 enumeration areas, consisting of 166 urban and 234 rural enumeration areas. All the households from the selected enumeration areas were listed in the second stage of sampling, from which individual households were to be selected. Households located in institutions such as army barracks, police camps, hospitals and boarding schools were omitted from the listing. These households represent a distinct distribution of the population mainly consisting of working males, aged between 20 and 50 years, demographics which are not representative of the entire Zimbabwean population. A random sample of 11,196 households was selected from the enumeration areas, which formed the final list for the 2015 ZDHS [3].

For this study, births that occurred 13–24 months before the survey for infants and those that occurred 60–119 months before the ZDHS were considered for the logistic regression analysis. This was done to ensure that only those equally fully exposed to the risk of death were analyzed to ascertain that differences were not brought about by censoring but by variables under study.

### Bias and confounding

During the 2015 ZDHS, interviewer bias was mitigated by the training of interviewers and by the use of translated structured interview questionnaires, and by the large response rate recorded from the survey, where 96.2% of all eligible women invited consented to participate in the ZDHS. In this study, matching spouses allowed improvement of data accuracy though corroboration of responses.

### Definition of variables

Infant and childhood mortality were the dependent variables. Independent variables were paternal age, smoking status, alcohol use, level of education, occupation, and income. Covariates that were controlled for were maternal age, maternal education, maternal health seeking behaviors, infant birth weight, and household wealth index.

The paternal factors were investigated for significance while controlling for biodemographic factors. The bio‑demographic factors were categorized to include especially maternal characteristics such as age of the mother at birth, birth order, preceding birth interval, and sex of the child. The age of the mother at birth was obtained by subtracting the child’s birth date and mother’s birth date, and coming up with three categories of maternal age: under 20 years (young), 20–39 years (old), and 40+ years (old). Order of birth was classed as 1, 2–4, and 5+. The preceding birth interval (in months) was classed into five, being first births, 9–23, 24–35, 36–47, 48–59, and 60+ on the basis of recommendations from the World Health Organization of an optimum birth interval of at least 24 months and methods used in previous studies [7, 8].

The variables that were used to locate husband and wife respondents for a particular case for infant and child mortality were household number (V002 and MV002) and head of household (V150 and MV150). The location of wife and husband was confirmed by comparing the line listing of spouse (V034 and MV034) for each of the respondents giving maternal, paternal, and infant/child data. Data that were discrepant in this regard were discarded to minimize misinformation.

### Statistical analysis

STATA 14 statistical package was used for the statistical analysis. Analysis of the data was conducted using univariate, bivariate, and multivariate statistical methods. Further bivariate analyses were conducted using the Kaplan–Meier and Cox test of equality for the survival data. The Cox proportional hazards model and logistic regression were performed for the infant and child survival data in the multivariate analyses separately. Kaplan–Meier survival curves were obtained for the combined data of children and infants.

The logistic regression analysis only considered those fully exposed to the risk of death within the period of interest. In this case, only births that occurred 13–24 months and 60–119 months before the survey for infant and child analyses, respectively, were considered for the logistic regression analysis to make sure that only those fully exposed were used in the analysis.

## Results

### Distribution of study population

A total of 5,722 eligible children and 3,887 fathers were identified for the study, where some fathers had more than one child. Each child’s case was treated independently, hence a sample size of 5,722 was used in the analysis. The under‑5‑year (U5) age distribution of deaths showed that the majority (almost 61%) of deaths occurred in children aged 1–5 years compared with mortality among neonates (Table 1). The sex of the infants and children was not significantly different, as was distribution according to province. The sample distribution by demographic characteristics of the fathers indicated that most of the deaths in infants and children occurred in rural areas as compared with urban areas (*p* = 0.000). The education status of the fathers indicated that most had at least reached secondary education. Fathers were mostly aged between 30 and 44 years of age, with a small number above the age of 50 years and below the age of 19 years, as shown in Table 1.

**Table 1:** Key demographics distribution of U5 mortality by age category, province, resident and gender (*n* = 5,772).

VARIABLE	CHILD IS ALIVE (FREQUENCY)		
	YES	NO	TOTAL
**Under‑5‑year mortality by age category**			
Neonates (< 28 days old)	461	84	545
Infants (28 days ≤ age ≤1 year)	992	353	1,345
Children (1 year < age ≤ 5 years)	3,136	746	3,882
**Total**	**4,589 (79.5%)**	**1,183 (20.5%)**	5,772 (100%)^*^
**Under‑5‑year mortality by province**			
Manicaland	558	189	747
Mashonaland Central	542	151	693
Mashonaland East	422	145	567
Mashonaland West	559	174	733
Matabeleland North	365	93	458
Matabeleland South	284	54	338
Midlands	543	125	668
Masvingo	495	112	607
Harare	541	99	640
Bulawayo	280	41	321
**Total**	**4,589 (79.5%)**	**1,183 (20.5%)**	5,772 (100%) *
**Under‑5‑year mortality by sex**			
Male	2,256	645	2,901
Female	2,333	538	2,871
**Total**	**4,589 (79.5%)**	**1,183 (20.5%)**	5,772 (100%) *
**Under‑5‑year mortality by geographical location**			
Urban	1,699	309	2,008
Rural	2,890	874	3,764
**Total**	**1,183 (20.5%)**	**1,183 (20.5%)**	5,772 (100%) *
**Father’s age group (years)**			
15–19			49
20–24			243
25–29			643
30–34			901
35–39			824
40–44			599
45–49			385
50–54			243
**Total**			3,887*
**Father’s level of education**			
No education			42
Primary			957
Secondary			2,358
Higher			530
**Total**			3,887*

*Total number of children (5,722) differed from the total number of fathers (3,887) due to a father/household having more than one eligible child. The sample size of 5,722 was used in the final analysis, as each child was treated independently.

### Logistic regression

Logistic regression was done to analyze the effect of the study covariates on the odds of whether a child lives or dies. In the bivariate logistic regression, the vital status of the child and individual covariates were regressed. As shown in Tables 2 and 3, the father’s age, residence/geographical location, educational level, wealth index, employment, and work category were found to be significant at α = 0.05, in both infant and child analyses. Infants and children with fathers who were older, were living in urban areas, were better educated, and had a better wealth index and employment status had better odds of survival compared with those with fathers who were younger, were living in rural areas, were uneducated, had a poor wealth index, and were unemployed.

**Table 2:** Bivariate logistic regression of paternal factors for infant mortality.

CHILD ALIVE	ODDS RATIO	*P* > Z	95% CONFIDENCE INTERVAL	
**Father’s age (years)**				
15–19	1.35	**0.000**	1.24	1.67
20–24	1.22	0.002	1.15	2.34
25–29	1.13	0.031	1.05	1.87
30–34	1.09	0.341	0.96	2.11
35–39	1.01	0.401	0.88	1.77
40–44	0.91	0.002	0.60	0.98
45–49	0.80	0.001	0.73	0.94
50–54	1	**–**	–	–
**Residence**				
Urban	1	**–**	–	–
Rural	1.31	**0.000**	1.18	1.45
**Paternal education**				
No education	1.36	**0.006**	1.13	1.74
Primary	1.13	0.041	1.04	2.31
Secondary	0.96	0.021	0.66	1.42
Higher	1	**–**	–	–
**Religion**				
Apostolic sect	1.22	0.087	.73	1.98
Christian	0.86	0.213	0.44	1.77
Other	1	–	–	–
**Wealth index**				
Poorer	1.29	0.014	1.10	2.09
Middle	1.11	0.023	0.91	1.60
Richer	0.87	**0.004**	0.79	0.96
Richest	1	**–**	–	–
**Father’s smoking**				
Smoker	1.13	0.295	0.90	1.41
Non‑smoker	1	–	–	–
**Employment statu**s				
Unemployed	1.76	**0.001**	1.32	1.94
Employed	1	**–**	–	–
**Work category**				
White collar	1	**–**	–	–
Blue collar	1.21	**0.031**	1.00	1.31
**Alcohol intake**				
Yes	1.11	0.468	0.84	1.47
No	1	–	–	–

**Table 3:** Bivariate logistic regression of paternal factors for child mortality.

CHILD ALIVE	ODDS RATIO	*P* > Z	95% CONFIDENCE INTERVAL	
**Father’s age (years)**				
15–19	1.35	**0.000**	1.24	1.48
20–24	1.22	0.001	1.02	1.78
25–29	1.13	0.021	1.05	1.65
30–34	1.11	0.022	1.01	2.31
35–39	1.08	0.041	0.98	1.33
40–44	0.90	0.004	0.40	0.98
45–49	0.78	0.011	0.68	0.95
50–54	1	**–**	–	–
**Paternal education**				
No education	1.18	**0.006**	1.01	1.95
Primary	1.11	0.021	1.03	1.33
Secondary	0.87	0.002	0.53	0.97
Higher	1	**–**	–	–
**Wealth index**				
Poorer	1.40	0.001	1.11	2.30
Middle	1.09	0.021	1.01	1.98
Richer	0.82	**0.025**	0.68	0.97
Richest	1	**–**	–	–
**Father’s smoking**				
Smoker	1.01	0.955	0.78	1.31
Non‑smoker	1	–	–	–
**Residence**				
Urban	1	**–**	–	–
Rural	1.86	**0.0021**	1.00	1.43
**Work category**				
White collar	1	**–**	–	–
Blue collar	1.01	**0.0028**	1.00	1.22
**Alcohol intake**				
Yes	1.13	0.478	0.81	1.56
No	1	–	–	–
**Religion**				
Apostolic sect	0.85	0.072	0.98	1.00
Christian	1	–	–	–
**Employment status**				
Unemployed	1.27	**0.0032**	1.19	2.03
Employed	1	**–**	–	–

After adjusting for maternal and birth characteristics, paternal geographical location, wealth index, and work category were significant in determining whether an infant lived or died within the first year of life at α = 0.05. Infants of fathers who were living in rural areas, had a poor wealth index, and were unemployed had higher odds of mortality compared with those of fathers living in urban areas, had a better wealth index, and were employed (Table 4).

**Table 4:** Adjusted logistic regression for infants.

VITAL STATUS OF CHILD/INFANT	ODDS RATIO	*P* > Z	[95% CONFIDENCE INTERVAL]	
**Maternal age**	1.23	**0.0158**	1.02	1.64
**Maternal education**	0.51	**0.026**	0.28	0.92
**Preceding birth interval**	0.98	0.034	0.97	1.00
**Birth size**	0.92	0.633	0.66	1.29
**Paternal geographical location (urban versus rural)**	0.78	**0.0016**	0.20	0.96
**Level of education**				
Primary	0.74	0.808	0.67	1.20
Secondary	0.60	0.686	0.50	1.17
Higher	1.56	0.742	0.11	2.09
**Wealth index**				
Poorer	1.52	**0.0081**	1.40	1.84
Middle	0.90	**0.0468**	0.27	0.98
Richer	0.94	**0.0420**	0.21	1.21
Richest	0.68	**0.0023**	0.21	0.83
**Religion**				
Apostolic	0.64	0.535	0.16	2.61
Christian	0.37	0.203	0.08	1.71
Other	0.52	0.371	0.12	2.20
**Work category**				
White collar	0.85	**0.0230**	0.37	0.96
Blue collar	1.92	0.179	1.31	2.69
**Age category**	1.05	0.892	0.49	2.25
**Smoking status**	1.29	0.532	0.58	2.82
**Alcohol intake**	1.98	0.095	0.89	2.40

The adjusted logistic regression for paternal factors yielded work category, age category, geographical location/place of residence, and wealth index as statistically significant predictors of child mortality (Table 5). Children of fathers working in white‑collar jobs had lower odds of death compared with those with unemployed fathers (odds ratio [OR]: 0.55; 95% confidence interval [CI]: 0.31–0.97; *p* = 0.038). Fathers that stayed in urban areas had children that had about a 30% reduction in the odds of childhood mortality compared with children whose fathers stayed in urban areas (OR: 0.73; 95% CI: 0.33–0.98; *p* = 0.4). The odds of death for children whose fathers received primary, higher, and secondary education was on average 40% lower when compared with those who did not receive any form of education, although this was not statistically significant. Poorer fathers had children with odds of death that were 40% higher than those who were poor (OR: 1.49; 95% CI: 1.15–1.56). The richest fathers had children with odds of death that were 10% lower.

**Table 5:** Adjusted logistic regression for children.

VITAL STATUS OF INFANT/CHILD	ODDS RATIO	*P* > Z	[95% CONFIDENCE INTERVAL]	
**Maternal age**	1.93	0.000	1.69	2.20
**Maternal education**	0.51	**0.000**	0.38	0.69
**Place of residence**	0.73	**0.004**	0.33	0.88
**Level of education**				
Primary	0.69	0.542	0.21	2.27
Secondary	.058	0.373	0.18	1.92
Higher	0.66	0.532	0.18	2.42
**Wealth index**				
Poorer	1.49	**0.002**	1.16	1.56
Poor	0.79	**0.021**	0.42	1.00
Middle	0.83	**0.033**	0.37	0.89
Richest	0.89	**0.0.001**	0.34	0.93
**Religion**				
Apostolic	1.29	0.575	0.53	3.10
Christian	1.30	0.564	0.53	3.16
Other	1.06	0.897	0.43	2.60
**Work category**				
White collar	0.55	**0.038**	0.31	0.97
Blue collar	0.68	0.114	0.41	1.10
**Age category**	1.09	0.678	0.75	1.54
**Smoking status**	1.22	0.404	0.77	1.94
**Alcohol intake**	0.93	0.710	0.63	1.38

### Survival analysis

**Figure 1:**
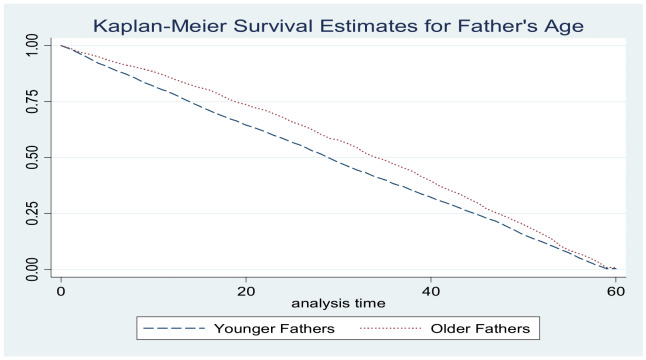
Kaplan–Meier survival estimates for father’s age.

**Figure 2:**
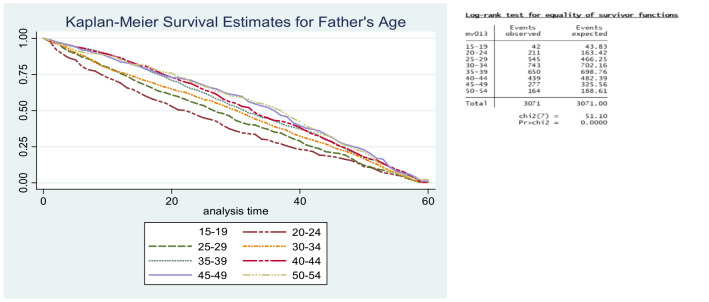
Kaplan–Meier survival estimates for father’s age group.

The smoking status of the father had no effect on the survival odds of infants and children. Both non‑smoking and smoking fathers had children with similar survival patterns. The difference in the two curves overall was found to be statistically insignificant. Concerning the father’s age, survival odds for children born to older fathers were higher than those born to younger fathers (*p* = 0.000; Figures 1 and 2).

In the first six months of life, the survival patterns were similar across different work categories. After this period, infants and children of those who are unemployed showed poor survival as compared with those from fathers working in blue‑collar and white‑collar jobs. This difference was only apparent until the 24 months of life, where survival patterns become similar. The survival of children and infants of fathers practicing traditional religion was better from 6 months until 48 months of life. Prior to and after this age category, the survival patterns were similar across the different religious groups. The Apostolic, Christian, and other religious groups of fathers had similar survival patterns. The survival patterns of children and infants were similar regardless of whether the father drank alcohol or not.

**Figure 3:**
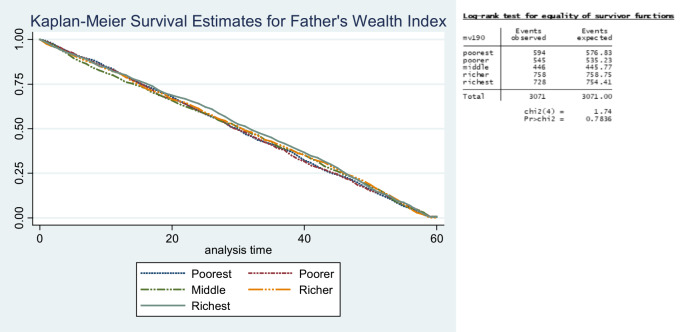
Survival estimate for father’s wealth index.

**Figure 4:**
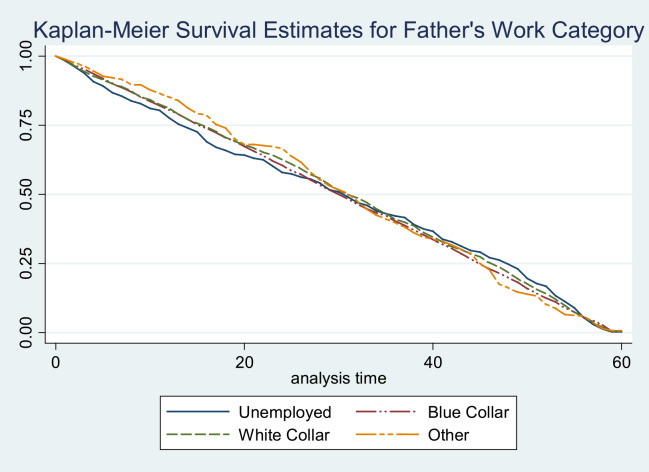
Survival estimate for father’s work category.

In the first 6 months of life, survival patterns were similar across the different wealth categories of fathers. After the 6 months, the children and infants of the poorest fathers started to show poor survival odds when compared with other wealth categories until the age of 24 months. From 18 months to 48 months, children of the richest fathers had better survival odds (Figures 3 and 4).

**Figure 5:**
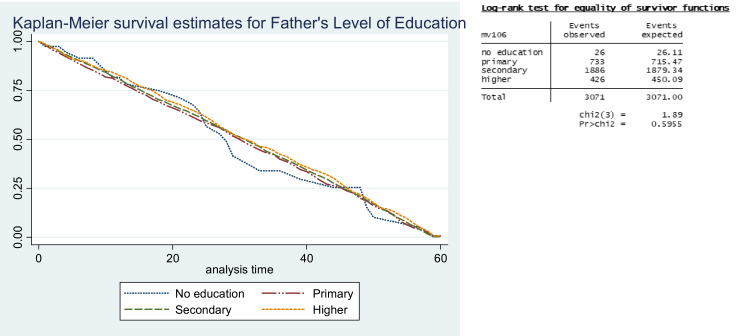
Survival estimates for father’s level of education.

Although the difference in curves failed to achieve statistical significance, the children of fathers with higher education had better survival when compared with other education categories (Figure 5). Infants and children staying in the urban areas had better survival as compared with those living in the rural areas.

## Discussion

The study assessed paternal social determinants for childhood mortality in Zimbabwe, and found that younger paternal age, a lower level of education, lower wealth index, unemployment, and a rural geographical location are important contributing paternal factors for childhood mortality, with notable effect sizes (based on confidence intervals) and practical significance (from logistic regression).

Considering paternal age, level of education, wealth index, employment, and geographical location that emerged (on the basis of effect size) to be important paternal factors contributing to shaping childhood mortality outcomes, the findings are relevant in the sense of social determinants of health. In low‑income contexts such as Zimbabwe, advanced paternal age is usually associated with greater financial stability and greater socioeconomic resources needed to provide better living conditions for children in their early lives than younger paternal age is [11]. A better level of education improves literacy levels required for making informed decisions regarding the child’s health and well‑being and increases opportunities for better employment or better income, which improves income or wealth index, and consequently, the power and control over the conditions of living, such as better housing, accessibility to healthcare, and nutrition, among others [12–14]. However, a lower level of education paints a bleak future, often linked to low literacy levels linked to misinformed decisions about children’s health and well‑being and lower chances of securing better‑paying employment, thereby resulting in low income and less power to control the conditions of living, predisposing children to lower survival chances [14]. Hence, children born to fathers with the best factors experience the best outcomes in the form of better survival, and those born to fathers with the poorest conditions face poorer survival outcomes, hence the old adage of good health outcomes for the richer and poor health outcomes for the poorer.

Paternal age was significant only for the child analysis and not the infant analysis, possibly because of the dominance of birth and maternal characteristics in determining mortality in the first year of life [14, 15]. Birth characteristics such as small birth weight are more important determinants at this stage, as they are related to birth complications and health problems such as cardiovascular diseases later in life, as informed by the Barker Theory of Fetal Origins of Adult Diseases [16].

Marked deviations were noted, such as the absence of statistical significance (based on *p*‑values) between some of the paternal social determinants and childhood mortality following adjustments to child and maternal‑specific factors when survival estimates were performed. There are two possible explanations for this:

- In the sense of social determinants of health, no one factor is more important than the other; rather, the factors interact and interdepend on each other to produce the observed outcomes [14, 15]. That paternal factors did not show statistical significance may not be because they are not significant/important in shaping childhood mortality outcomes, but rather because the observed outcomes are not caused by paternal factors alone. Hence, unadjusted calculations were statistically significant. For instance, the more consistently higher the household income is, the higher the chances of children from that household surviving will be because the parents are more likely to be able to afford providing the children with a healthier environment or better living conditions to live in, such as better healthcare, better housing, and a safe and nutritious diet, among others. On the contrary, the inverse is true for children from lower‑income households, whose survival chances are likely to be lower and bleaker. In that sense, it is the size of the total disposable income that matters, and whatever contribution comes will make the income higher, and this may improve child survival chances. Hence, there might not be a statistically significant result between a component or a contributory factor and the outcome, not because it is not important but because, alone, it is not sufficient to produce a measurable outcome. More to the point, paternal income alone may not produce any significant observable changes to child survival outcomes, but through interacting with other factors, such as parental level of education, age, religion, geographical location and macro‑level issues such as global, national policies, and maternal and child characteristics [12–15]. In this case, social determinants for childhood survival include several factors at the individual (birth characteristics), household (paternal and maternal), community, and public/global policy level, and these factors interconnect and interact with each other in producing survival outcomes. Our study therefore substantiates that statistical significance can be inconclusive about the importance of factors, and that no one factor is more important than the other in producing outcomes.
- Though total population data were used, the sample size used in the study was not “planned” or statistically determined according to some justified level of alpha or beta particularly for the study. The *p*‑value is in essence arbitrary, having no good cutoffs to it and, hence, needs to be interpreted relative to the research context [17]. Moreover, the existing data may have been unsuited to detect childhood mortality exclusively attributable to parental factors—we did not manipulate the data in any way to increase the chances of generating significant results, such as through removal of outliers. Future studies are required to scrutinize pertinent issues such as data quality (data accuracy, authenticity, and objectivity), required sample size, and how suited the data are for studying paternal determinants of childhood survival (in terms of data scales, presence of outliers, and variable definition).

## Limitations of the Study

- Use of the total population sampling approach and use of existing data may have resulted in inadequate and unsuited data for determining effect size in survival estimates.
- There was no comparison between survival outcomes for children living with their fathers and those living with single mothers, to further substantiate our study findings.

## Conclusion

Paternal characteristics are important contributing factors for child survival, but they may not be sufficient alone. The interaction between child characteristics, household (paternal and maternal), community, and public/global‑policy‑level factors is important in shaping observed childhood mortality outcomes. Social determinants for child survival are interlinked and interdependent on each other in producing the observed childhood mortality outcomes, and no one factor is more important than the other. Each factor represents an important component but is not individually sufficient when it comes to producing an outcome.

## Data Availability

The data analyzed during the current study are available from the corresponding author [Dr. Laston Gonah, Email: lgonah@wsu.ac.za] on reasonable request to bona fide researchers.
